# Copper Conundrum: Navigating Atypical Wilson's Disease Through Radiological Insights

**DOI:** 10.7759/cureus.65203

**Published:** 2024-07-23

**Authors:** Devyansh Nimodia, Pratapsingh Parihar, Roohi G Gupta, Sakshi S Dudhe, Prasad Desale, Shubhi Gaur

**Affiliations:** 1 Radiodiagnosis, Jawaharlal Nehru Medical College, Datta Meghe Institute of Higher Education and Research, Wardha, IND

**Keywords:** ceruloplasmin, copper metabolism, magnetic resonance imaging (mri), atp-7b gene, wilson's disease

## Abstract

Wilson's disease is a rare genetic disorder characterized by abnormal copper metabolism due to mutations in the *ATP-7B* gene. This case report details the presentation of a 14-year-old male child exhibiting severe generalized dystonia, rigidity, myoclonic jerks, dysarthria, and excessive salivary drooling. During ophthalmic examination, Kayser-Fleischer rings were identified. Symmetrical hyperintensities in cortical and subcortical areas, including the basal ganglia and brainstem, were noted on brain magnetic resonance imaging (MRI). Additionally, diffusion restriction in the bilateral fronto-parietal region was observed. The diagnosis of Wilson's disease was confirmed through further diagnostic assessments, such as serum ceruloplasmin levels and urine copper excretion. Treatment was initiated with penicillamine, anticonvulsants, and supportive measures, resulting in partial recovery after a three-month follow-up period. This case emphasizes the significance of identifying atypical MRI brain findings in Wilson's disease, which aids in early diagnosis and appropriate management to prevent irreversible neurological damage.

## Introduction

Wilson's disease is an infrequent, autosomal recessive, congenital abnormality in the metabolism of copper in individuals, which can be traced back to the mutation in ATPase copper-transporting beta (ATP7B) gene locus on the long arm of Chromosome 13. This gene is responsible for the synthesis of ceruloplasmin, a copper protein transporter [1]. The cause of the disease is unknown, but it is believed to be due to abnormal processing of ceruloplasmin in the liver. Defects lead to protease-resistant ceruloplasmin in the blood and bile. Neurologic symptoms of Wilson's disease are caused by copper accumulation in the brain damaging nerve cells [2]. The clinical presentation of Wilson's disease typically occurs between the ages of 5 and 50. However, in early childhood cases, the disease usually manifests as chronic liver disease or hemolytic anemia, and neurological symptoms are rare before the age of 10 [3]. Metabolic disorders like Wilson's disease can manifest as hepatitis, hepatic decompensation, or cirrhosis. Neurological symptoms often appear in the early twenties. In some cases, hepatic issues may not show noticeable symptoms, leading to neurologic complaints being the first clinical sign [4]. We introduce a pediatric patient who presented with rigidity, myoclonic jerks, and dystonia and was subsequently diagnosed with Wilson's disease [4]. Radiologists are familiar with common abnormalities in the basal ganglia and brainstem in Wilson's disease. Along with well-known basal ganglia lesions, there can be extensive gray and white matter lesions. A case report discusses a child with Wilson's disease showing abnormalities in both gray and white matter, along with a brief literature review [3]. Although the magnetic resonance imaging (MRI) findings of the brain in adult Wilson's disease have been extensively documented in the literature, the depiction of MRI findings in childhood Wilson's disease remains scarce. The MRI of the patient's brain revealed uncommon manifestations of symmetrical signal changes in the white matter.

## Case presentation

**Figure 1:**
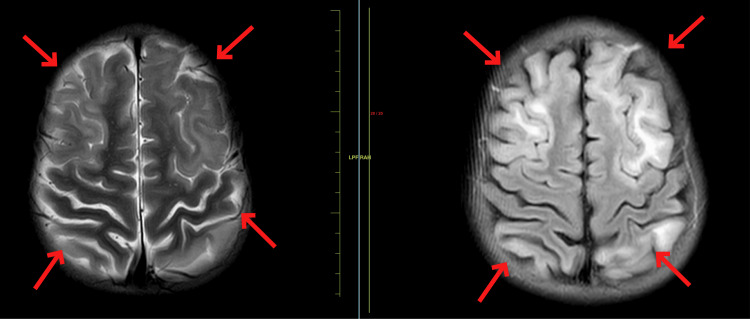
MRI showing T2/FLAIR hyperintensities in cortical and subcortical areas of bilateral fronto-parietal lobes. MRI: Magnetic resonance imaging; FLAIR: fluid-attenuated inversion recovery

**Figure 2:**
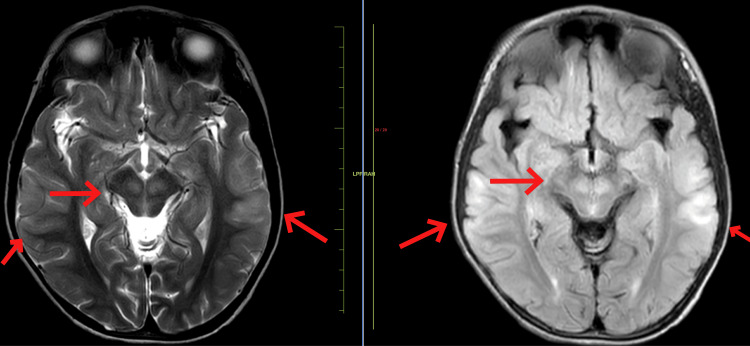
MRI showing T2/FLAIR symmetrical hyperintensities noted in the bilateral temporal lobe with sparing of occipital lobes. MRI: Magnetic resonance imaging; FLAIR: fluid-attenuated inversion recovery

**Figure 3:**
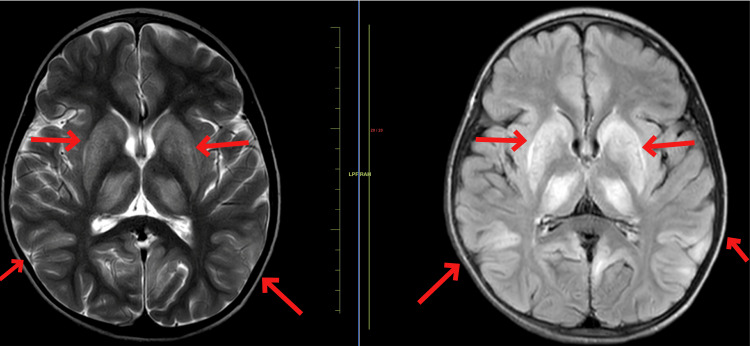
MRI showing T2/FLAIR symmetrical hyperintensities noted in the bilateral temporal lobe with sparing of occipital lobes. T2/FLAIR symmetrical hyperintensities noted in bilateral basal ganglia including corpus striatum, putamen, and thalamus.⁠ MRI: Magnetic resonance imaging; FLAIR: fluid-attenuated inversion recovery

**Figure 4:**
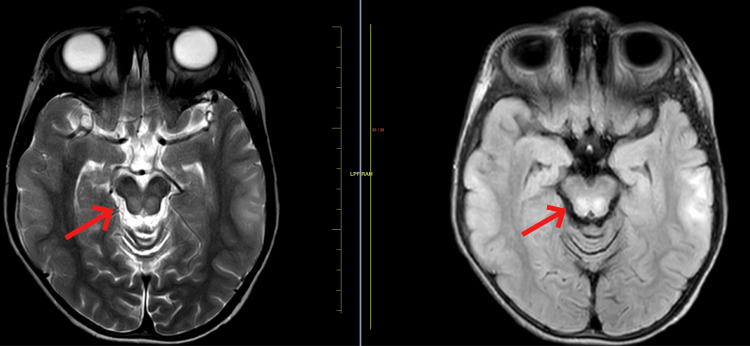
MRI showing T2/FLAIR hyperintense focal lesions noted in brain stem including tegmentum of the midbrain. MRI: Magnetic resonance imaging; FLAIR: fluid-attenuated inversion recovery

**Figure 5:**
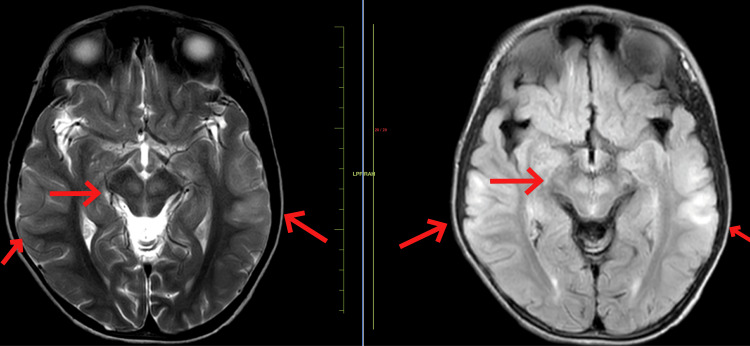
MRI showing T2/FLAIR hyperintensities noted in bilateral red nucleus, superior and inferior colliculus and periaqueductal region. MRI: Magnetic resonance imaging; FLAIR: fluid-attenuated inversion recovery

**Figure 6:**
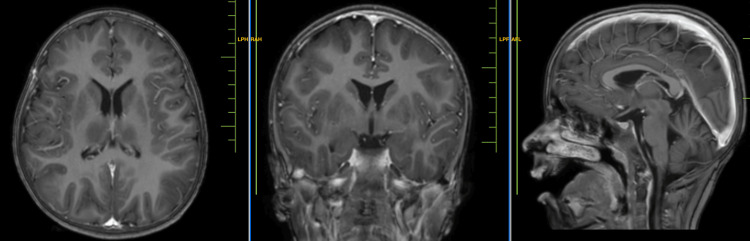
Contrast-enhanced magnetic resonance imaging (CE-MRI) showing no abnormal enhancement.

A 14-year-old male child presented to the medicine department with complaints of severe generalized dystonia over the past four months. Upon examination, the child was conscious, cooperative, and oriented to time, place, and person. He had a normal birth and immunization history. The patient had normal vitals and normal heart and breath sounds. The patient started having complaints of abnormal positioning of his lower limbs four months ago which led to difficulty in walking and the patient suffered multiple falls. Soon after that, he developed abnormal positioning of his hand which led to difficulty in doing day-to-day chores. These abnormal positioning worsened and led to severe dystonic posturing of the entire body rendering the patient bedridden for the last two months. The patient also complained of dysarthria and excessive saliva drooling, severe painful spasms in his limbs, and mood changes over the past month. Upon ophthalmic examination, the child had Kayser-Fleischer (KF) rings. Upon the history given by the patient and his parents and our examination, we sought the possibility of a post-infective demyelinating disease affecting the central nervous system. Routine blood investigations were done which came out normal. ⁠A magnetic resonance imaging (MRI) scan of the brain was performed, and it revealed T2/FLAIR hyperintense areas noted in bilateral cortical and subcortical areas of the bilateral cerebral hemisphere, predominantly in bilateral fronto-parietal in Figure 1 and temporal lobes with sparing of the occipital region (Figure 2) showing diffusion restriction with a corresponding low signal on ADC. ⁠T2/FLAIR symmetrical hyperintensities were also noted in bilateral basal ganglia including corpus striatum, putamen, and thalamus (Figure 3). ⁠Similar T2/FLAIR hyperintense focal lesions were noted in the brain stem including the tegmentum of the midbrain in Figure 4, bilateral red nucleus, superior and inferior colliculus, periaqueductal region in Figure 5, and superior peduncle at the level of pons on both the sides in Figure 5. No enhancement was obtained in post-contrast study in Figure 6.

A cerebrospinal fluid study (CSF) was done which revealed no evidence of any infectious cause. An electroencephalogram (EEG) was performed on which episodes of abnormal electrical activity in the form of paroxysmal spike and wave epileptiform discharges were obtained. We came to a possible differential diagnosis of a neurometabolic disorder with rapid decompensation due to the presence of cognitive decline, myoclonus, subcortical involvement on brain imaging, and normal cerebrospinal fluid (CSF) findings. Further laboratory investigations revealed low serum ceruloplasmin levels, high 24-hour urine copper excretion, and low serum copper levels (Table 1).

**Table 1:** Laboratory investigations

Laboratory Investigations	Value	Normal Range
Serum ceruloplasmin level	8.5 mg/dl	18-35mg/dl
24-hour urine copper excretion	460 μg	20-35 μg
Serum copper	57.9 μg/dl	70-140 μg/dl

Additional investigation of ultrasonography of the abdomen and pelvis was done which was reported normal. Although the patient presented with cognitive decline, myoclonus, and exanthematous fever, the high 24-hour urine copper excretion and the absence of periodicity in the epileptiform discharges on EEG made the differential diagnosis of subacute sclerosing panencephalitis unlikely. A diagnosis of Wilson's disease was made using radiological and laboratory investigations, and treatment with penicillamine 750mg/day, along with anticonvulsants and other supportive measures, was initiated. Following the diagnostic evaluation, the patient was advised to take gradually increasing doses of D-penicillamine, with a daily dosage of 250 mg at the time of discharge, aimed at preventing sudden exacerbation of neurological symptoms. Additionally, zinc acetate at a dosage of 100 mg three times a day and pyridoxine were included in the treatment regimen. Notably, there was no observed deterioration in the neurological symptoms throughout the course of treatment, and the patient did not manifest any adverse reactions to the prescribed medications. Subsequently, family members of the patient were screened and were reported normal. The patient was discharged from the hospital and a follow-up appointment was scheduled to monitor the progress and adjust the treatment plan as necessary. This comprehensive approach highlights the importance of continuous monitoring and individualized treatment strategies in the management of Wilson's disease across different age groups. 

## Discussion

The levels of serum ceruloplasmin, the copper protein transporter, are notably reduced, and there is an increased excretion of copper in the urine. However, it is important to note that the most definitive method of diagnosing Wilson's disease is by estimating the quantity of copper present in the liver [5]. Therefore, a comprehensive evaluation of the liver's copper content must be conducted in order to confirm the diagnosis. In conclusion, Wilson's disease, known as hepatolenticular degeneration, is an inherited disorder that disrupts the metabolism of copper in various bodily systems. This condition is caused by a mutation in the copper adenosine triphosphate transporter (ATP 7B) gene, which impairs the synthesis of ceruloplasmin, the copper protein transporter [6]. As a result, the liver is unable to eliminate copper effectively, leading to an excessive buildup of copper primarily in the liver and the brain [2]. Wilson's disease presents with a wide range of clinical manifestations, affecting the liver, nervous system, eyes, psychiatric well-being, and other systems. While reduced serum ceruloplasmin levels and increased copper excretion in the urine are indicative of the disease, the most conclusive method of diagnosis involves assessing the amount of copper in the liver. It is crucial for healthcare professionals to conduct a thorough examination of the liver's copper content to confirm the presence of Wilson's disease [5]. Histopathological studies have revealed abnormalities throughout the central nervous system in individuals with Wilson's disease. These abnormalities include formation of cavities, softening with a spongy texture, a general reduction in the number of neurons, the presence of Opalski cells, increased cellularity, and atrophy [7]. It is believed that these pathological changes result from an excessive amount of extracellular copper, which induces oxidative stress and leads to the destruction of cells [4]. Neuroimaging studies have demonstrated a wide range of abnormalities in individuals with Wilson's disease [3]. The most characteristic findings include areas of increased signal intensity on T2-weighted sequences in the lentiform nuclei, followed by involvement of the caudate nuclei, thalami, midbrain, and pons. These findings are likely due to demyelination, softening, spongy formation, and cavitation [8]. Three main white matter tracts affected in Wilson's disease are dentatorubrothalamic, pontocerebellar, and corticospinal tracts. The dentatorubral tract starts at the dentate nucleus, goes through the superior cerebellar peduncle, and red nucleus, and ends in the ventrolateral thalamic nucleus. The pontocerebellar tract originates from pontine nuclei and extends to various cerebellar lobules through the middle cerebellar peduncle. These tracts are part of the cortico-ponto-cerebello-dentatorubro-thalamo-cortical circuit in the extrapyramidal system. Wilson's disease often causes abnormalities due to the involvement of the extrapyramidal grey nuclei. The corticospinal (pyramidal) tract consists of projection fibers from the cerebral cortex, passing through various brain regions and reaching the spinal cord [9]. Subcortical white matter abnormalities situated in the frontal, parietal, and temporal lobes, manifesting as diminished signal intensity on T1-weighted scans and heightened signal intensity on T2-weighted scans, have been recorded among individuals diagnosed with Wilson's disease [10]. Moreover, cerebral atrophy, as evidenced by ventricular enlargement particularly in the frontal horns, and cerebellar atrophy are similarly detected in this cohort [11]. The correlation between neuroimaging findings and the progression of clinical symptoms has not yet been established. Brain MRIs frequently display hyperintensities in the lentiform and caudate nucleus, thalamus, and midbrain in patients with Wilson's disease, excluding the red nucleus and substantia nigra [5]. These increased signals on T2-weighted images are usually the result of edema, gliosis, or cystic degeneration [12]. In some instances, hyperintensities are also noted in long transgressive-regressive sequences, which may be due to the paramagnetic effects of copper accumulation [13]. While diffusion restriction in MRI may occasionally be observed as a result of cytotoxic edema or inflammation, it is generally not seen in chronic cases [13]. Extensive white matter lesions were detected in the bilateral frontal and parietooccipital regions of the individual under examination, manifesting a symmetrical pattern, a phenomenon that is seldom documented in the neuroimaging literature concerning patients with Wilson's disease. Furthermore, there was an observation of diffusion restriction in the bilateral fronto-parietal region, a characteristic often linked to acute instances, notwithstanding the fact that our subject had been experiencing symptoms for a duration of four months. We present a case of Wilson's disease in a child with basal ganglia lesions that showed significant involvement of white matter tracts. This disease occurs in about 1 in 30,000-40,000 people in most populations. Neurological symptoms linked to this disorder include dystonia, incoordination, tremor, and dysarthria [6]. White matter abnormalities in MRI brain scans of patients with Wilson's disease are rarely reported. The diagnosis of Wilson's disease was considered based on the clinical assessment and laboratory findings. The patient underwent penicillamine and zinc therapy, along with dietary modifications. Dystonia medications were also administered. After a follow-up period of three months, the patient showed partial recovery. Repeat laboratory findings indicated normalization of values. The current case is presented in order to emphasize the existence of unusual MRI brain observations in Wilson's disease, a condition that can be severe yet responsive to treatment: 1. Bilateral cortical and subcortical regions of the cerebral hemispheres exhibit symmetrical white matter hyperintensities, predominantly in the fronto-parietal and temporal lobes, while sparing the occipital region. Furthermore, involvement is seen in bilateral basal ganglia such as the corpus striatum, putamen, and thalamus, as well as in the tegmentum of the midbrain, bilateral red nucleus, superior and inferior colliculus, periaqueductal region, and superior peduncle at the pons level on both sides and 2. In a patient with a four-month medical history, diffusion restriction is evident in the bilateral fronto-parietal region, potentially suggesting an ongoing metabolic injury to the brain. An MRI scan of the brain was performed and it revealed areas noted appearing T2/FLAIR hyperintense showing DWI restriction with corresponding low signal on ADC. ⁠T2/FLAIR symmetrical hyperintensities were also noted. ⁠Similar T2/FLAIR hyperintense focal lesions were noted in the brain stem. No enhancement was obtained in the post-contrast study. The potential differential diagnoses for these white matter alterations encompass conditions such as HIV encephalopathy, extrapontine myelinolysis, acute disseminated encephalomyelitis, and posthypoxic encephalopathy, all of which were meticulously excluded through a thorough review of the patient's medical background and investigative procedures. While cases of Wilson's disease have been known to present with cerebral and cerebellar atrophy along with ventricular dilatation, this particular patient did not exhibit such radiological features, as indicated by the medical records. In sum, the complexity of the neuroimaging findings, in this case, adds to the existing body of knowledge on the diagnostic challenges posed by white matter changes and emphasizes the importance of a comprehensive approach to ruling out differential diagnoses in patients with similar presentations [14].

## Conclusions

This case study provides a detailed depiction of a pediatric patient with severe generalized dystonia diagnosed with Wilson's disease. Neuroimaging revealed white matter abnormalities and basal ganglia lesions, showcasing varied neurological symptoms. Diagnosis involved clinical assessment, laboratory tests, and neuroimaging for timely treatment. The case underscores the significance of considering Wilson's disease in the differential diagnosis of pediatric patients exhibiting neurological symptoms, especially in the presence of atypical features. Moreover, it highlights the importance of neuroimaging in revealing the underlying pathological changes and guiding treatment strategies. Research on clinical presentations and neuroimaging in Wilson's disease will enhance understanding. Awareness among healthcare providers is crucial for early detection and intervention in Wilson's disease, improving patient outcomes.
